# Variations in Influenza Vaccination by Clinic Appointment Time and an Active Choice Intervention in the Electronic Health Record to Increase Influenza Vaccination

**DOI:** 10.1001/jamanetworkopen.2018.1770

**Published:** 2018-09-14

**Authors:** Rebecca H. Kim, Susan C. Day, Dylan S. Small, Christopher K. Snider, Charles A. L. Rareshide, Mitesh S. Patel

**Affiliations:** 1 Penn Medicine Nudge Unit, University of Pennsylvania, Philadelphia; 2 Perelman School of Medicine, University of Pennsylvania, Philadelphia; 3 Wharton School, University of Pennsylvania, Philadelphia; 4 Crescenz Veterans Affairs Medical Center, Philadelphia, Pennsylvania

## Abstract

**Importance:**

Influenza vaccination rates in the United States are suboptimal near 40%, but little is known about variations in care based on clinic appointment time.

**Objectives:**

To compare differences in influenza vaccination rates by clinic appointment time and to evaluate the association of an active choice intervention in the electronic health record with changes in vaccination rates.

**Design, Setting, and Participants:**

Retrospective, quality improvement study of 11 primary care practices at the University of Pennsylvania Health System from September 1, 2014, to March 31, 2017. Participants included adults eligible for influenza vaccination. Data analysis was conducted from October 20, 2017, to March 9, 2018.

**Interventions:**

During the 2016 to 2017 influenza season, 3 primary care practices at the University of Pennsylvania Health System implemented an active choice intervention in the electronic health record that prompted medical assistants to ask patients about influenza vaccination during check-in and template vaccination orders for clinicians to review during the visit.

**Main Outcomes and Measures:**

Influenza vaccination rates.

**Results:**

The sample comprised 96 291 patients with a mean (SD) age of 56.2 (17.0) years; 41 865 (43.5%) were men, 61 813 (64.2%) were white, and 23 802 (24.7%) were black. Among all practices across all 3 years, vaccination rates were approximately 44% from 8 am to 10 am, declined to 41.2% by 11 am and 38.3% at noon, increased to 40.2% at 1 pm, and then declined to 34.3% at 3 pm and 32.0% at 4 pm (*P* < .001 for adjusted linear trend). For the 3 years, vaccination rates were 46.9%, 47.2%, and 45.6% at control practices and 49.7%, 52.2%, and 59.3% at intervention practices, respectively. In adjusted analyses, compared with control practices over time, the active choice intervention was associated with a significant 9.5–percentage point increase in vaccination rates (95% CI, 4.1-14.3; *P* < .001). Vaccination rates increased similarly across times of the day.

**Conclusions and Relevance:**

Influenza vaccination rates significantly declined as the clinic day progressed. The active choice intervention was associated with a significant increase in influenza vaccination rates that were similar in magnitude throughout the day.

## Key Points

**Question:**

Do influenza vaccination rates vary by clinic appointment time and can an active choice intervention in the electronic health record directed to medical assistants improve vaccination rates in primary care practices?

**Findings:**

In this quality improvement study of 11 primary care practices and 96 291 patients, influenza vaccination rates significantly declined as the clinic day progressed. Primary care practices that implemented an active choice intervention in the electronic health record to prompt medical assistants to ask patients about influenza vaccination and template vaccination orders for clinicians to review were associated with a significant increase in influenza vaccination rates when compared with a control group of primary care practices.

**Meaning:**

Reminders in the electronic health record can improve vaccination rates overall, but other strategies may also be needed to address variations by time of day.

## Teaser

This quality improvement study compares differences in influenza vaccination rates by clinic appointment time and evaluates whether an active choice intervention in the electronic health record is associated with changes in vaccination rates.

## Introduction

Influenza is a significant cause of illness, hospitalization, and death in the United States.^1^ While influenza vaccination has been demonstrated to reduce the burden of disease,^2,3^ more than 50% of adults in the United States do not receive the vaccine each year.^4^ The Centers for Disease Control and Prevention recommend that clinicians offer patients the vaccine during clinic visits.^5^ However, primary care physicians (PCPs) often fall behind schedule as the clinic day progresses and this tendency may lead to rushed visits, decision fatigue, and suboptimal care. For example, inappropriate antibiotic prescribing rates have been shown to be greater later in the clinic day.^6^ To our knowledge, there has been no evaluation of variations in vaccination rates by clinic appointment time.

Nudges are changes to choice architecture that can have outsized effects on medical decision making.^7^ For example, in prior work we found that using active choice, a method that requires clinicians to accept or decline an influenza vaccination order in the electronic health record (EHR), significantly increased vaccination rates at 1 primary care practice.^8^ However, this type of approach could lead to clinician alert fatigue.^9,10,11^ So before expanding to 3 other practices at our institution, the active choice intervention was redirected to medical assistants who could ask patients about influenza vaccination and template orders in the EHR for clinicians to review. If variations in care exist by clinic appointment time, these types of nudges could address them. The objective of this study was to evaluate differences in influenza vaccination rates by clinic appointment time and compare changes in vaccination rates among practices receiving an active choice intervention in the EHR with those among control practices.

## Methods

The University of Pennsylvania institutional review board approved this study and waived informed consent because it was infeasible given the study design, and the study posed minimal risk. This study followed the Standards for Quality Improvement Reporting Excellence (SQUIRE) reporting guideline.

### Setting and Participants

The sample comprised adult patients at 11 primary care practices at the University of Pennsylvania Health System who had a new or return patient clinic visit with their PCP during at least 1 of 3 influenza seasons (September 1 to March 31) between 2014 and 2017. The intervention practice sites included 3 internal medicine clinics in Philadelphia and a suburb that chose to adapt evidence from our pilot work^8^ to implement the intervention described in this study. Control practices included 8 internal medicine and family medicine clinics in Philadelphia and the suburbs. These control practices were selected through the following process. First, we identified all primary care practices at our main primary care network at the University of Pennsylvania Health System. We included both internal medicine practice (who belonged to the same division as the intervention practices but decided not to implement the intervention) and family medicine practices (who belonged to a different division and did not discuss the option of implementing the intervention). Second, since preliminary data indicated that the overall vaccination rate during the preintervention period at these 22 control practices were much lower (approximately 30%) than the intervention practices (approximately 50%), we selected practices that were within a 10–percentage point range above or below the average of the intervention sites (approximate range, 40%-60%) and had at least 500 eligible patients in all 3 influenza seasons. This provided a more comparable control group of 8 practices with sufficient sample sizes at each practice. Practice sites’ locations, specialties, and vaccination rates in each of the influenza seasons for the 3 intervention and 8 control practices are available in eTable 1 in the Supplement.

To compare vaccination rates by clinic appointment time, we evaluated patients at their first visit with their PCP in each season. Patients whose first PCP visit was not a new or return visit were excluded since vaccination may not be appropriate for acute, sick, or other visits (n = 15 440). Patients who were already vaccinated based on EHR documentation were also excluded (n = 29 692) (eFigure 1 in the Supplement).

To compare changes in vaccination rates in practices with and without the active choice intervention, we evaluated patients during entire influenza seasons. Patients who switched PCPs within a season (n = 446), did not have a PCP visit (n = 7857), did not have a new or return visit with their PCP at any point during the season (n = 1624), or were already vaccinated based on EHR documentation were excluded (n = 30 024) (eFigure 2 in the Supplement).

### Intervention

Prior to the intervention, PCPs had to remember to manually check if a patient was due for influenza vaccination, discuss it with the patient, and then place an order for it in the EHR. During the 2016 to 2017 influenza season, 3 University of Pennsylvania Health System primary care practices implemented an active choice intervention in the EHR using a best practice alert in EPIC directed to medical assistants (eFigure 3 in the Supplement). Prior to meeting with the clinician, patients met with a medical assistant to check their vitals. At that time, the EHR checked for patient eligibility for the influenza vaccine and prompted medical assistants to accept or cancel an order for the vaccine. If accepted, the order would be templated for the clinician to review and sign during the patient visit. This intervention was similar in design to prior work,^8,12^ except that it was delivered only to medical assistants and not to clinicians.

### Data

Clarity, an EPIC reporting database, was used to obtain data on patients (demographics, insurance, comorbidities, prior influenza vaccination status, and PCPs) and clinic visits (date, appointment time, practice site, visit type, and presence of an order for influenza vaccination or not). Health insurance claims data were not available for this study. However, in prior work,^8^ more than 99.9% of vaccine orders also had an insurance claim indicating almost all were both ordered and completed. Household income level was obtained using the US Census data on median household income based on zip code.

### Statistical Analysis

The primary outcome measure was the percentage of eligible patients who had the influenza vaccination ordered. To evaluate vaccination rates by clinic appointment time, we grouped appointment times by each hour (eg, appointments for 8 am, 8:15 am, 8:30 am, and 8:45 am were all grouped to 8 am). In the adjusted analysis of patient visit level data, we used PROC GENMOD to fit the model based on generalized estimating equations with a logit link and an independence correlation structure using PCPs as the clustering unit.^13^ The model was adjusted for patient demographics (age, sex, race/ethnicity, and household income), insurance, Charlson comorbidity index,^14^ practice group (intervention or control site), clinic visit type (new or return), fixed effects by practice site, year and calendar month, and a covariate for each appointment hour. To obtain an adjusted linear trend of vaccination as the clinic day progressed, the same model was fit, but instead of covariates for each appointment hour, we used a continuous variable for appointment time (from 8 am to 4 pm).

To evaluate the association of changes in vaccination rates with the active choice intervention, we used a difference-in-differences approach.^15,16^ Similar to prior work,^8,12^ we compared changes in vaccination order rates at the intervention vs control practices during the postintervention year relative to the 2 preintervention years. We used the patient as the unit of analysis and included all clinic visits during each influenza season. Since patients were not always represented in all 3 years and some switched PCPs, patients who appeared in the data for multiple years were treated as independent patients for each year as was done in prior work.^8,12^ The same generalized estimating equations model was fit for analysis of patient season level data but without visit level covariates (appointment time or visit type) and with interaction terms for practice group (intervention vs control) and year. To obtain the adjusted difference in the percentage of patients vaccinated along with 95% confidence intervals, we used the bootstrap procedure, resampling patients 1000 times.^17,18^ Resampling of patients was conducted by PCPs to maintain clustering at the PCP level. A test of controls was performed to test the null hypothesis of parallel trends between the intervention and control practices during the 2 preintervention years.^19^ We conducted several sensitivity analyses with different modeling structures. First, we fit the difference-in-differences model, also adjusting for the number of patient visits with their PCP in each season. Second, we fit a generalized linear mixed model using PCP random effects nested under practice random effects. Two-sided hypothesis tests used a significance level of .05; all analyses were conducted in SAS, version 9.4 (SAS Institute Inc).

## Results

The sample of the entire influenza seasons comprised 96 291 patients with a mean (SD) age of 56.2 (17.0) years; 41 865 (43.5%) were men, 61 813 (64.2%) were white, and 23 802 (24.7%) were black (Table 1). The sample of first visits with the PCP comprised 72 908 (87.3%) return patients and 10 585 (12.7%) new patients (eTable 2 in the Supplement).

**Table 1.** Sample Characteristics for Patients Visiting With Their Primary Care Physician Any Time During the Entire Influenza Season^a^

Characteristic	No. (%) by Influenza Season						
	2014-2015		2015-2016		2016-2017		Total, All Years
	Control	Intervention	Control	Intervention	Control	Intervention	
Patients, No.	20 994	10 152	21 399	11 155	22 864	9727	96 291
Age, mean (SD), y	56.2 (17.4)	55.0 (16.0)	56.6 (17.4)	55.9 (16.1)	56.7 (17.4)	56.0 (16.2)	56.2 (17.0)
Male	9305 (44.3)	4351 (42.9)	9385 (43.9)	4645 (41.6)	10 089 (44.1)	4090 (42.0)	41 865 (43.5)
Race/ethnicity							
White non-Hispanic	14 449 (68.8)	5558 (54.7)	14 833 (69.3)	6028 (54)	15 821 (69.2)	5124 (52.7)	61 813 (64.2)
Black non-Hispanic	4549 (21.7)	3270 (32.2)	4418 (20.6)	3692 (33.1)	4559 (19.9)	3314 (34.1)	23 802 (24.7)
Asian	369 (1.8)	493 (4.9)	382 (1.8)	512 (4.6)	445 (1.9)	461 (4.7)	2662 (2.8)
Hispanic	388 (1.8)	120 (1.2)	401 (1.9)	144 (1.3)	398 (1.7)	130 (1.3)	1581 (1.6)
Other/unknown	1239 (5.9)	711 (7.0)	1365 (6.4)	779 (7.0)	1641 (7.2)	698 (7.2)	6433 (6.7)
Insurance							
Private	12 880 (61.4)	6460 (63.6)	12 924 (60.4)	6657 (59.7)	13 911 (60.8)	5572 (57.3)	58 404 (60.7)
Medicare	7057 (33.6)	2953 (29.1)	7265 (34.0)	3519 (31.5)	7819 (34.2)	3167 (32.6)	31 780 (33.0)
Medicaid	1057 (5.0)	739 (7.3)	1210 (5.7)	979 (8.8)	1134 (5.0)	988 (10.2)	6107 (6.3)
Annual household income, $^b^							
<50 000	4651 (22.2)	3799 (37.4)	4470 (20.9)	4268 (38.3)	4572 (20.0)	3885 (39.9)	25 645 (26.6)
50 000-100 000	13 806 (65.8)	3860 (38.0)	14 230 (66.5)	4213 (37.8)	15 265 (66.8)	3568 (36.7)	54 942 (57.1)
>100 000	2359 (11.2)	2436 (24.0)	2529 (11.8)	2621 (23.5)	2847 (12.5)	2221 (22.8)	15 013 (15.6)
Missing	178 (0.8)	57 (0.6)	170 (0.8)	53 (0.5)	180 (0.8)	53 (0.5)	691 (0.7)
Charlson comorbidity index, median (IQR)	0 (0-2)	1 (0-2)	1 (0-2)	1 (0-3)	1 (0-2)	1 (0-3)	1 (0-2)

Abbreviation: IQR, interquartile range.

^a^ Data represents characteristics of patients who had a new or return visit with their primary care physician any time during the influenza season from September to March.

^b^ Annual household income was linked to each patient using the US Census data on median household income based on zip code.

**Figure 1.**
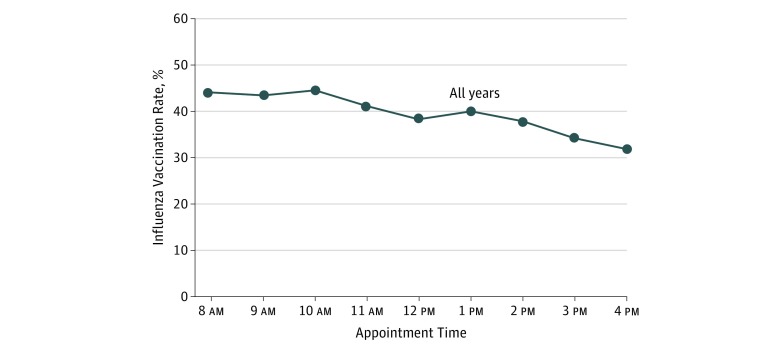
Influenza Vaccination Rates by Clinic Appointment Time for All Practices in All 3 Years Vaccination rates are based on each patient’s first visit with his or her primary care physician. Appointment times are grouped by the start of the hour (eg, 8:15 am and 8:30 am were grouped to 8 am).

Figure 1 displays the unadjusted influenza vaccination rates by clinic appointment time for all practices during all 3 seasons. Vaccination rates were approximately 44% from 8 am to 10 am and then declined to 41.2% at 11 am and 38.3% at noon. Vaccination rates increased slightly to 40.2% at 1 pm and then declined to 34.3% at 3 pm and 32.0% at 4 pm. Relative to the 8 am appointment time, the adjusted odds ratios of vaccination were significantly lower for each subsequent hour of the day and for the overall linear trend (adjusted odds ratio, 0.95; 95% CI, 0.94-0.96; *P* < .001) (Table 2). The distribution of patients by clinic appointment time in year 3 was similar among patients vaccinated and not vaccinated in year 2 (eTable 3 in the Supplement), indicating that patients who historically received the vaccine did not make earlier appointments than those who did not receive the vaccine in the past. Regression tables for the models are available in eTables 4 and 5 in the Supplement.

**Table 2.** Adjusted Odds of Influenza Vaccination by Appointment Time

Appointment Time	Vaccination Relative to 8 am Appointment Time, Adjusted OR (95% CI)^a^	*P* Value
8 am	1 [Reference]	NA
9 am	0.92 (0.85-0.98)	.01
10 am	0.89 (0.82-0.98)	.01
11 am	0.77 (0.70-0.86)	<.001
12 pm	0.68 (0.57-0.80)	<.001
1 pm	0.79 (0.72-0.87)	<.001
2 pm	0.78 (0.72-0.85)	<.001
3 pm	0.67 (0.61-0.74)	<.001
4 pm	0.62 (0.54-0.70)	<.001
Overall time trend^b^	0.95 (0.94-0.96)	<.001

Abbreviations: NA, not applicable; OR, odds ratio.

^a^ Adjusted ORs represent the relative odds of vaccination for each additional hour after 8 am. Appointment times are grouped by the start of the hour (eg, 8:15 am and 8:30 am were grouped into 8 am).

^b^ Overall time trend uses an adjusted model with a continuous variable for appointment time with 1 equal to 8 am and 9 equal to 4 pm. The ORs represents the relative odds of vaccination for each incremental 1-hour period. For example, an OR of 0.95 can be interpreted at 5% lower odds per hour for each hour after 8 am.

**Figure 2.**
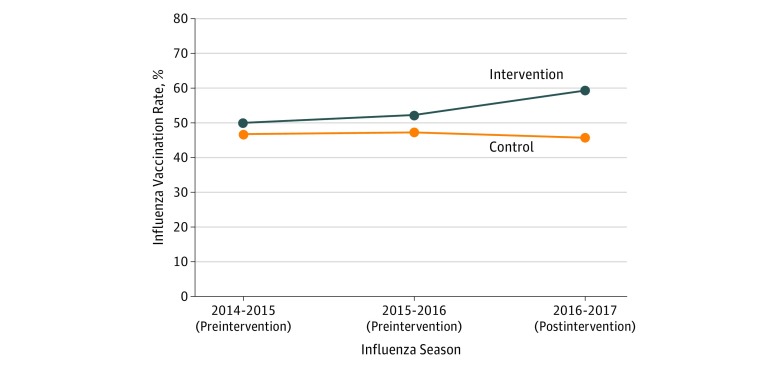
Influenza Vaccination Rates by Practice Group and Year Vaccination rates include all patient visits at the 3 intervention practices and 8 control practices. The active choice intervention was implemented at the intervention practices during the 2016 to 2017 influenza season.

Figure 2 displays the unadjusted influenza vaccination rate by control and intervention practice group by year. For the 3 years, vaccination rates were 46.9%, 47.2%, and 45.6% at control practices and 49.7%, 52.2%, and 59.3% at intervention practices, respectively. Adjusted preintervention trends during the first 2 years did not differ between groups (adjusted odds ratio, 1.08; 95% CI, 0.92-1.27; *P* = .36). In adjusted analyses, there was a significant 9.5–percentage point increase (95% CI, 4.1-14.3; *P* < .001) in influenza vaccination rates for the intervention practices relative to the compared practices over time. The regression table for the model is available in eTable 6 in the Supplement. Sensitivity analyses using different modeling structures had similar findings (eTables 7 and 8 in the Supplement).

**Figure 3.**
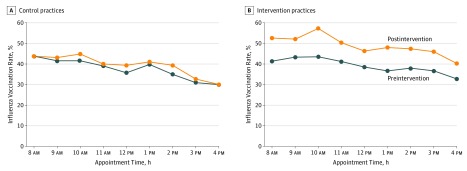
Influenza Vaccination Rates by Clinic Appointment Time Before and After an Active Choice Intervention in the Electronic Health Record Vaccination rates are based on each patient’s first visit with his or her primary care physician. Appointment times are grouped by the start of the hour (eg, 8:15 am and 8:30 am were grouped to 8 am). Preintervention represents the first influenza seasons from 2014 to 2016. Postintervention represents the third influenza season from 2016 to 2017. A, Control practices were not exposed to the intervention in either period. B, Intervention practices were exposed during the postintervention period.

Figure 3 displays unadjusted influenza vaccination rates for control and intervention during the first 2 years (preintervention) and the third year (postintervention). Among control practices, vaccination trends were similar in both periods but rates were 0 to 5 percentage points higher in the postintervention period (Figure 3A). Among intervention practices, vaccination trends were similar in both periods, but rates were 8 to 14 percentage points greater in the postintervention period (Figure 3B). Among intervention practices, vaccination rates at the end of the day (4 pm) were 33% in the preintervention period and 40% in the postintervention period, which were nearly the same as at 8 am in the preintervention period (41%).

## Discussion

Among a network of primary care practices, we found that influenza vaccination rates significantly declined as the clinic day progressed. An active choice intervention delivered to medical assistants through the EHR was associated with a significant 9.5–percentage point increase in influenza vaccination rates, which is nearly a 20% relative increase compared with the preintervention period. However, even after the intervention, influenza rates declined as the clinic day progressed. To our knowledge, this is one of the first studies of its kind to evaluate variations in influenza vaccination rates by clinic appointment time. Our findings demonstrate the potential of using nudges in the EHR to improve vaccination rates, but further strategies may be needed to address variations in care by time of day.

These findings expand our understanding of how practice environments influence medical decision making in several ways. First, time of day may play an important role in influencing patient care. This could be because as the clinic day progresses, clinicians often fall behind schedule, leading to rushed visits, or because clinicians may be susceptible to decision fatigue, the depletion of self-control and active initiative that results from the cumulative burden of making decisions.^20^ These tendencies have been demonstrated in other areas of health care. Linder and colleagues^6^ examined behaviors at 23 primary care practices and found that physicians had higher rates of inappropriate antibiotic prescriptions as clinic sessions progressed. Dai and colleagues^21^ evaluated health care workers at 35 hospitals and found that hand hygiene compliance rates declined as time progressed through a work shift. Both Linder et al and Dai et al found that when health care professionals were given a break in the middle of the day or shift, behaviors temporarily improved but then continued to progressively worsen. We also found that influenza vaccination rates increased from noon to 1 pm, presumably after a lunch break for the clinicians and staff, but then continued to decline over the course of the rest of the day. It is important to note that practice- or patient-level factors may also play important roles in medical decision-making behaviors over the course of the clinic day. Future research could further evaluate the relative contribution of clinicians, medical assistants, and other staff, practice workflow, and patient-level factors on vaccination rates. Medical decision-making behaviors over the course of clinic day should also be evaluated in other settings and for other behaviors.

Second, as medical decision making is increasingly occurring within digital environments, the design and framing of information in the EHR can influence clinician behaviors. We found that an active choice intervention that prompted medical assistants to make decisions on whether to template vaccinations orders in the EHR for clinicians to review was associated with a significant increase in vaccination rates. These findings add to the existing literature on using nudges to increase influenza vaccination. Milkman and colleagues^22^ conducted a randomized clinical trial at a large employer and found that participants who were asked to plan a date and time for the vaccination had a 4.2–percentage point increase in vaccination rates when compared with control. Chapman and colleagues^23^ randomly assigned university employees to receive an email with an opt-in vs opt-out framing for influenza vaccination and found that those with the opt-out default setting had a 12–percentage point increase in vaccination rates compared with control. Our study differs from these 2 in that it was directed to clinicians rather than patients, was conducted in primary care practices, and leveraged a scalable approach through the EHR. Future research could compare nudges that are targeted with clinicians, patients, or both in combination.

Third, the design of the active choice intervention differed in this study compared with our previous study and reveals important insights. In prior work, the intervention was delivered to physicians and medical assistants.^8^ While it was associated with a 6.6–percentage point increase in vaccination rates, physicians reported alert fatigue and there was confusion between whether the medical assistant or physician was responsible for addressing the alert. Therefore, before expanding the approach to other practices, the decision was made to deliver the alert only to medical assistants. This design increased clarity in who was responsible for addressing the alert and reduced the burden on physicians. While the 2 studies were conducted in different practices, it is important to highlight that the intervention in this study achieved a greater percentage point increase in vaccination rates (9.5 vs 6.6) on a much higher baseline preintervention level (51% vs 18%). Future research could compare the effectiveness of active choice interventions and other types of nudges such as setting EHR defaults through randomized clinical trials and in other settings.

### Limitations

This study had limitations. First, any observational study is susceptible to unmeasured confounders. However, the active choice intervention was evaluated using a difference-in-differences approach, which reduces potential bias from unmeasured cofounders by comparing changes in vaccination levels over time between the intervention and control practices. Second, this study was conducted within a single health system, which may limit generalizability. However, we included 11 practice sites from 2 different states. Nonetheless, findings should be confirmed in other settings. Third, changes in vaccination rates by clinic appointment time may be owing to physician, practice, and patient factors and we were not able to evaluate their relative contribution. However, our multivariate models do adjust for available physician, practice, and patient characteristics.

## Conclusions

Influenza vaccination rates significantly declined as the clinic day progressed. The active choice intervention was associated with a significant increase in vaccination rates that were similar in magnitude throughout the day. These findings indicate that time of day and choice architecture play an important role in influencing clinician behaviors and patient outcomes. Scalable approaches that leverage the EHR to deploy well-designed nudges may be a promising approach to improve medical decision-making behaviors, but further work is needed to address variations in vaccination rates by time of day.

## Notes

Supplement. **eTable 1.** Practice Site Location, Specialty, and Annual Vaccination Rates **eTable 2.** Sample Characteristics for Patients At Their First Visit With their Primary Care Physician **eTable 3.** Vaccination Rates and Distribution by Clinic Appointment Time in Year 3 (2016-2017) Among Patients That Were Vaccinated or Not in Year 2 (2015-2016) **eTable 4.** Regression Table for Clinic Appointment Time Model With Hourly Appointment Time Variables **eTable 5.** Regression Table for Clinic Appointment Time Model With Continuous Appointment Time Variable **eTable 6.** Regression Table for Difference-in-Differences Model **eTable 7.** Regression Table for Difference-in-Differences Model Also Adjusted for Number of Patients Visits With the PCP in Each Season **eTable 8.** Regression Table for Difference-in-Differences Model Using Generalized Linear Mixed Effects With Physician Random Effects Nested Within Practice Random Effects **eFigure 1.** Exclusions for the Patient Sample for the Seasonal Evaluation **eFigure 2.** Exclusions for the Patient Visit Sample to Define the First Visit With the Primary Care Physician **eFigure 3.** Active Choice Intervention Delivered Through a Best Practice Alert in EPIC

## References

[1] Centers for Disease Control and Prevention Disease burden of influenza. https://www.cdc.gov/flu/about/disease/burden.htm. Accessed April 1, 2018.

[2] Bridges CB, Thompson WW, Meltzer MI, Effectiveness and cost-benefit of influenza vaccination of healthy working adults: a randomized controlled trial. JAMA. 2000;284(13):-. doi:10.1001/jama.284.13.1655 11015795

[3] Nichol KL, Wuorenma J, von Sternberg T Benefits of influenza vaccination for low-, intermediate-, and high-risk senior citizens. Arch Intern Med. 1998;158(16):1769-1776. doi:10.1001/archinte.158.16.1769 9738606

[4] Centers for Disease Control and Prevention Influenza vaccination coverage. https://www.cdc.gov/flu/fluvaxview/index.htm. Accessed April 1, 2018.

[5] Grohskopf LA, Sokolow LZ, Broder KR, Prevention and control of seasonal influenza with vaccines: recommendations of the Advisory Committee on Immunization Practices—United States, 2017-18 influenza season. MMWR Recomm Rep. 2017;66(2):1-20. doi:10.15585/mmwr.rr6602a1 28841201 PMC5837399

[6] Linder JA, Doctor JN, Friedberg MW, Time of day and the decision to prescribe antibiotics. JAMA Intern Med. 2014;174(12):2029-2031. doi:10.1001/jamainternmed.2014.5225 25286067 PMC4648561

[7] Patel MS, Volpp KG, Asch DA Nudge units to improve the delivery of health care. N Engl J Med. 2018;378(3):214-216. doi:10.1056/NEJMp1712984 29342387 PMC6143141

[8] Patel MS, Volpp KG, Small DS, Using active choice within the electronic health record to increase influenza vaccination rates. J Gen Intern Med. 2017;32(7):790-795. doi:10.1007/s11606-017-4046-6 28337690 PMC5481246

[9] Black AD, Car J, Pagliari C, The impact of eHealth on the quality and safety of health care: a systematic overview. PLoS Med. 2011;8(1):e1000387. doi:10.1371/journal.pmed.1000387 21267058 PMC3022523

[10] van der Sijs H, Aarts J, Vulto A, Berg M Overriding of drug safety alerts in computerized physician order entry. J Am Med Inform Assoc. 2006;13(2):138-147. doi:10.1197/jamia.M1809 16357358 PMC1447540

[11] Avery AJ, Savelyich BS, Sheikh A, Identifying and establishing consensus on the most important safety features of GP computer systems: e-Delphi study. Inform Prim Care. 2005;13(1):3-12. 15949170 10.14236/jhi.v13i1.575

[12] Patel MS, Volpp KG, Small DS, Using active choice within the electronic health record to increase physician ordering and patient completion of high-value cancer screening tests. Healthc (Amst). 2016;4(4):340-345. doi:10.1016/j.hjdsi.2016.04.005 28007228 PMC7240802

[13] Zeger SL, Liang KY, Albert PS Models for longitudinal data: a generalized estimating equation approach. Biometrics. 1988;44(4):1049-1060. doi:10.2307/2531734 3233245

[14] Charlson ME, Pompei P, Ales KL, MacKenzie CR A new method of classifying prognostic comorbidity in longitudinal studies: development and validation. J Chronic Dis. 1987;40(5):373-383. doi:10.1016/0021-9681(87)90171-8 3558716

[15] Dimick JB, Ryan AM Methods for evaluating changes in health care policy: the difference-in-differences approach. JAMA. 2014;312(22):2401-2402. doi:10.1001/jama.2014.16153 25490331

[16] Shadish WR, Cook TD, Campbell DT Experimental and Quasi-Experimental Designs for Generalized Causal Inference. Boston, MA: Houghton Mifflin; 2001.

[17] Efron B, Tibshirani R An Introduction to the Bootstrap. New York, NY: Chapman & Hall; 1993. doi:10.1007/978-1-4899-4541-9

[18] Davison AC, Hinkley DV Bootstrap Methods and Their Application. New York, NY: Cambridge University Press; 1997. doi:10.1017/CBO9780511802843

[19] Meyer BD Natural and quasi-experiments in economics. J Bus Econ Stat. 1995;13(2):151-161.

[20] Vohs KD, Baumeister RF, Schmeichel BJ, Twenge JM, Nelson NM, Tice DM Making choices impairs subsequent self-control: a limited-resource account of decision making, self-regulation, and active initiative. J Pers Soc Psychol. 2008;94(5):883-898. doi:10.1037/0022-3514.94.5.883 18444745

[21] Dai H, Milkman KL, Hofmann DA, Staats BR The impact of time at work and time off from work on rule compliance: the case of hand hygiene in health care. J Appl Psychol. 2015;100(3):846-862. doi:10.1037/a0038067 25365728

[22] Milkman KL, Beshears J, Choi JJ, Laibson D, Madrian BC Using implementation intentions prompts to enhance influenza vaccination rates. Proc Natl Acad Sci U S A. 2011;108(26):10415-10420. doi:10.1073/pnas.1103170108 21670283 PMC3127912

[23] Chapman GB, Li M, Colby H, Yoon H Opting in vs opting out of influenza vaccination. JAMA. 2010;304(1):43-44. doi:10.1001/jama.2010.892 20606147

