# Supplementary material for: Variations in Influenza Vaccination by Clinic Appointment Time and an Active Choice Intervention in the Electronic Health Record to Increase Influenza Vaccination
Source: JAMA Netw Open. 2018 Sep 14;1(5):e181770. doi: 10.1001/jamanetworkopen.2018.1770 (PMC6324515; doi:10.1001/jamanetworkopen.2018.1770)
Supplement: Supplement. — eTable 1. Practice Site Location, Specialty, and Annual Vaccination Rates eTable 2. Sample Characteristics for Patients At Their First Visit With their Primary Care Physician eTable 3. Vaccination Rates and Distribution by Clinic Appointment Time in Year 3 (2016-2017) Among Patients That Were Vaccinated or Not in Year 2 (2015-2016) eTable 4. Regression Table for Clinic Appointment Time Model With Hourly Appointment Time Variables eTable 5. Regression Table for Clinic Appointment Time Model With Continuous Appointment Time Variable eTable 6. Regression Table for Difference-in-Differences Model eTable 7. Regression Table for Difference-in-Differences Model Also Adjusted for Number of Patients Visits With the PCP in Each Season eTable 8. Regression Table for Difference-in-Differences Model Using Generalized Linear Mixed Effects With Physician Random Effects Nested Within Practice Random Effects eFigure 1. Exclusions for the Patient Sample for the Seasonal Evaluation eFigure 2. Exclusions for the Patient Visit Sample to Define the First Visit With the Primary Care Physician eFigure 3. Active Choice Intervention Delivered Through a Best Practice Alert in EPIC [file jamanetwopen-1-e181770-s001.pdf]

Supplementary Online Content

Kim RH, Day SC, Small DS, Snider CK, Rareshide CAL, Patel MS. Variations in influenza vaccination by clinic appointment time and an active choice intervention in the electronic health record to increase influenza vaccination. *JAMA Netw Open*. 2018;1(5):e181770. doi:10.1001/jamanetworkopen.2018.1770

eTable 1. Practice Site Location, Specialty, and Annual Vaccination Rates

eTable 2. Sample Characteristics for Patients At Their First Visit With their Primary Care Physician

eTable 3. Vaccination Rates and Distribution by Clinic Appointment Time in Year 3 (2016-2017) Among Patients That Were Vaccinated or Not in Year 2 (2015-2016)

eTable 4. Regression Table for Clinic Appointment Time Model With Hourly Appointment Time Variables

eTable 5. Regression Table for Clinic Appointment Time Model With Continuous Appointment Time Variable

eTable 6. Regression Table for Difference-in-Differences Model

eTable 7. Regression Table for Difference-in-Differences Model Also Adjusted for Number of Patients Visits With the PCP in Each Season

eTable 8. Regression Table for Difference-in-Differences Model Using Generalized Linear Mixed Effects With Physician Random Effects Nested Within Practice Random Effects

eFigure 1. Exclusions for the Patient Sample for the Seasonal Evaluation

eFigure 2. Exclusions for the Patient Visit Sample to Define the First Visit With the Primary Care Physician

eFigure 3. Active Choice Intervention Delivered Through a Best Practice Alert in EPIC

This supplementary material has been provided by the authors to give readers additional information about their work.

© 2018 Kim RH et al. *JAMA Network Open*.

eTable 1. Practice Site Location, Specialty, and Annual Vaccination Rates

Practice Group	Name	Location	Specialty	% Vaccinated, 2014-2015	% Vaccinated, 2015-2016	% Vaccinated, 2016-2017
**Intervention Sites**	Penn Internal Medicine University City	Philadelphia, PA	Internal medicine	47.2%	52.7%	58.5%
	Penn Center for Primary Care	Philadelphia, PA	Internal medicine	50.7%	58.0%	62.7%
	Penn Internal Medicine Radnor	Radnor, PA	Internal medicine	51.1%	47.2%	57.6%
**Control Sites**	Penn Internal Medicine Cirigliano Practice	Philadelphia, PA	Internal medicine	58.1%	64.3%	64.4%
	Penn Internal Medicine Media	Media, PA	Internal medicine	51.3%	49.2%	50.6%
	Penn Internal Medicine J. Edwin Wood Clinic	Philadelphia, PA	Internal medicine	49.3%	53.0%	63.8%
	Penn Family Medicine Kennett	Kennet Square, PA	Family medicine	43.5%	43.9%	45.5%
	Penn Medicine Bala Cynwyd	Bala Cynwyd, PA	Internal medicine and Family medicine	48.3%	46.9%	41.0%
	Penn Internal Medicine Woodbury Heights	Woodbury Heights, NJ	Internal medicine	41.5%	41.7%	35.3%
	Penn Family Medicine Unionville	Kennet Square, PA	Family medicine	39.9%	39.4%	46.0%
	Penn Presbyterian Internal Medicine	Philadelphia, PA	Internal medicine and Family medicine	42.6%	49.1%	41.4%

© 2018 Kim RH et al. *JAMA Network Open*.

eTable 2. Sample Characteristics for Patients At Their First Visit With their Primary Care Physician

Characteristic	2014-2015		2015-2016		2016-2017		All Years
	Control	Intervention	Control	Intervention	Control	Intervention	Total
Patients, n	17816	9645	18083	10174	18826	8949	83493
Mean age (SD), y	56.5 (17.2)	55.3 (16.1)	56.9 (17.2)	56.0 (16.1)	57.0 (17.2)	56.4 (16.3)	56.5 (16.9)
Male sex, n (%)	7998 (44.9)	4095 (42.5)	7893 (43.6)	4239 (41.7)	8438 (44.8)	3802 (42.5)	36465 (43.7)
Race/ethnicity, n (%)							
White non-hispanic	11857 (66.6)	5403 (56)	12179 (67.4)	5664 (55.7)	12688 (67.4)	4832 (54.0)	52623 (63.0)
Black non-hispanic	4247 (23.8)	3008 (31.2)	4087 (22.6)	3191 (31.4)	4084 (21.7)	2929 (32.7)	21546 (25.8)
Asian	336 (1.9)	459 (4.8)	338 (1.9)	481 (4.7)	373 (2.0)	419 (4.7)	2406 (2.9)
Hispanic	325 (1.8)	115 (1.2)	319 (1.8)	132 (1.3)	305 (1.6)	126 (1.4)	1322 (1.6)
Other/Unknown	1051 (5.9)	660 (6.8)	1160 (6.4)	706 (6.9)	1376 (7.3)	643 (7.2)	5596 (6.7)
Insurance, n (%)							
Private	10754 (60.4)	6154 (63.8)	10757 (59.5)	6163 (60.6)	11366 (60.4)	5137 (57.4)	50331 (60.3)
Medicare	6050 (34.0)	2842 (29.5)	6212 (34.4)	3181 (31.3)	6505 (34.6)	2973 (33.2)	27763 (33.3)
Medicaid	1012 (5.7)	649 (6.7)	1114 (6.2)	830 (8.2)	955 (5.1)	839 (9.4)	5399 (6.5)
Annual household income, n (%)**							
Less than $50,000	4391 (24.6)	3516 (36.5)	4191 (23.2)	3708 (36.4)	4134 (22)	3437 (38.4)	23377 (28)
$50,000 to $100,000	11366 (63.8)	3765 (39.0)	11651 (64.4)	3946 (38.8)	12283 (65.2)	3366 (37.6)	46377 (55.5)
Greater than $100,000	1919 (10.8)	2305 (23.9)	2120 (11.7)	2476 (24.3)	2285 (12.1)	2101 (23.5)	13206 (15.8)
Missing	140 (0.8)	59 (0.6)	121 (0.7)	44 (0.4)	124 (0.7)	45 (0.5)	533 (0.6)

© 2018 Kim RH et al. *JAMA Network Open*.

Charlson comorbidity index, Median (IQR)	1 (0-2)	1 (0-2)	1 (0-2)	1 (0-2)	1 (0-2)	1 (0-3)	1 (0-2)
Clinic visits, n (%)							
New patient	1891 (10.6)	1408 (14.6)	2269 (12.5)	1532 (15.1)	2459 (13.1)	1026 (11.5)	10585 (12.7)
Return patient	15925 (89.4)	8237 (85.4)	15814 (87.5)	8642 (84.9)	16367 (86.9)	7923 (88.5)	72908 (87.3)

Abbreviations: SD=standard deviation; IQR=interquartile range.

*Data represents characteristics of patients at their first new or return visit with their primary care physician during the influenza season from September to March.

**Annual household income was linked to each patient using United States Census Data on median household income based on zip code

© 2018 Kim RH et al. *JAMA Network Open*.

eTable 3. Vaccination Rates and Distribution by Clinic Appointment Time in Year 3 (2016-2017) Among Patients That Were Vaccinated or Not in Year 2 (2015-2016)

Vaccinated in Prior Year of 2015-2016				
**Time**	**# Vaccinated**	**# Patients**	**% Vaccinated**	**% of Total Visits**
8AM	675	885	76.3%	13.1%
9AM	834	1125	74.1%	16.6%
10AM	862	1221	70.6%	18.1%
11AM	609	872	69.8%	12.9%
12PM	174	245	71.0%	3.6%
1PM	455	673	67.6%	10.0%
2PM	509	762	66.8%	11.3%
3PM	362	600	60.3%	8.9%
4PM	218	376	58.0%	5.6%
Total	4698	6759	69.5%	100.0%
**NOT Vaccinated in Prior Year of 2015-2016**				
**Time**	**# Vaccinated**	**# Patients**	**% Vaccinated**	**% of Total Visits**
8AM	154	770	20.0%	13.0%
9AM	192	1002	19.2%	17.0%
10AM	187	904	20.7%	15.3%
11AM	158	720	21.9%	12.2%
12PM	25	180	13.9%	3.0%
1PM	124	534	23.2%	9.0%
2PM	140	695	20.1%	11.8%
3PM	135	673	20.1%	11.4%
4PM	65	431	15.1%	7.3%
	1180	5909	20.0%	100.0%

© 2018 Kim RH et al. *JAMA Network Open*.

eTable 4. Regression Table for Clinic Appointment Time Model With Hourly Appointment Time Variables

Variable	Odds Ratio	P-Value
Influenza season		
Year 1	Ref	
Year 2	1.22	<.0001
Year 3	1.37	<.0001
Practice group		
Control	Ref	
Intervention	0.68	0.0001
Patient age in years	1.01	<.0001
Patient sex		
Male	Ref	
Female	0.91	0.004
Patient race/ethnicity		
White non-hispanic	Ref	
Asian/East Indian	1.09	0.13
Black non-hispanic	0.82	<.0001
Hispanic	0.97	0.69
Other	0.87	<.0001
Patient insurance		
Private	Ref	
Medicare	1.33	<.0001
Medicaid	1.29	<.0001
Patient charlson comorbidity index	1.01	0.003
Patient median household income	1.00	1.00
Intervention practice site		
Penn Internal Medicine Radnor	Ref	
Penn Internal Medicine University City	1.19	0.14
Penn Center for Primary Care	1.33	0.02
Control practice site		
Penn Internal Medicine Cirigliano Practice	Ref	
Penn Internal Medicine Media	0.55	<.0001
Penn Internal Medicine J. Edwin Wood Clinic	0.60	<.0001
Penn Family Medicine Kennett	0.70	0.007
Penn Medicine Bala Cynwyd	0.75	0.03
Penn Internal Medicine Woodbury Heights	0.54	<.0001
Penn Family Medicine Unionville	0.25	<.0001
Penn Presbyterian Internal Medicine	0.65	<.0001
Month		
September	Ref	

© 2018 Kim RH et al. *JAMA Network Open*.

October	2.25	<.0001
November	1.90	<.0001
December	1.02	0.81
January	0.51	<.0001
February	0.26	<.0001
March	0.04	<.0001
Visit Type		
Return patient visit	Ref	
New patient visit	0.84	0.0003
Clinic Appointment Time		
8am	Ref	
9am	0.92	0.01
10am	0.89	0.01
11am	0.77	<.0001
12pm	0.68	<.0001
1pm	0.79	<.0001
2pm	0.78	<.0001
3pm	0.67	<.0001
4pm	0.62	<.0001
Intercept	0.44	<.0001

*GEE Model is clustered by primary care physician

**Main outcome measure highlighted in blue

© 2018 Kim RH et al. *JAMA Network Open*.

eTable 5. Regression Table for Clinic Appointment Time Model With Continuous Appointment Time Variable

Variable	Odds Ratio	P-Value
Influenza season		
Year 1	Ref	
Year 2	1.22	<.0001
Year 3	1.37	<.0001
Practice group		
Control	Ref	
Intervention	0.69	0.0002
Patient age in years	1.01	<.0001
Patient sex		
Male	Ref	
Female	0.91	0.003
Patient race/ethnicity		
White non-hispanic	Ref	
Asian/East Indian	1.09	0.12
Black non-hispanic	0.82	<.0001
Hispanic	0.98	0.73
Other	0.87	<.0001
Patient insurance		
Private	Ref	
Medicare	1.33	<.0001
Medicaid	1.29	<.0001
Patient charlson comorbidity index	1.01	0.004
Patient median household income	1.00	0.01
Intervention practice site		
Penn Internal Medicine Radnor	Ref	
Penn Internal Medicine University City	1.19	0.14
Penn Center for Primary Care	1.33	0.02
Control practice site		
Penn Internal Medicine Cirigliano Practice	Ref	
Penn Internal Medicine Media	0.55	<.0001
Penn Internal Medicine J. Edwin Wood Clinic	0.61	<.0001
Penn Family Medicine Kennett	0.71	0.01
Penn Medicine Bala Cynwyd	0.76	0.04
Penn Internal Medicine Woodbury Heights	0.55	<.0001
Penn Family Medicine Unionville	0.25	<.0001
Penn Presbyterian Internal Medicine	0.66	<.0001
Month		
September	Ref	

© 2018 Kim RH et al. *JAMA Network Open*.

October	2.25	<.0001
November	1.90	<.0001
December	1.02	0.80
January	0.51	<.0001
February	0.27	<.0001
March	0.04	<.0001
Visit Type		
Return patient visit	Ref	
New patient visit	0.84	0.0008
Clinic Appointment Time as Continuous Variable	0.95	<.0001
Intercept	0.45	<.0001

*GEE Model is clustered by primary care physician

**Main outcome measure highlighted in blue

© 2018 Kim RH et al. *JAMA Network Open*.

eTable 6. Regression Table for Difference-in-Differences Model

Variable	Odds Ratio	P-Value
Influenza season		
Year 1	Ref	
Year 2	0.99	0.86
Year 3	0.94	0.83
Practice group		
Control	Ref	
Intervention	0.64	0.12
Patient age in years	1.02	<.0001
Patient sex		
Male	Ref	
Female	0.97	0.39
Patient race/ethnicity		
White non-hispanic	Ref	
Asian/East Indian	1.09	0.04
Black non-hispanic	0.86	<.0001
Hispanic	1.04	0.49
Other	0.81	<.0001
Patient insurance		
Private	Ref	
Medicare	1.61	<.0001
Medicaid	1.56	<.0001
Patient charlson comorbidity index	1.05	<.0001
Patient median household income	1.00	1.00
Intervention practice site		
Penn Internal Medicine Radnor	Ref	
Penn Internal Medicine University City	1.12	0.61
Penn Center for Primary Care	1.27	0.28
Control practice site		
Penn Internal Medicine Cirigliano Practice	Ref	
Penn Internal Medicine Media	0.62	0.02
Penn Internal Medicine J. Edwin Wood Clinic	0.88	0.56
Penn Family Medicine Kennett	0.67	0.16
Penn Medicine Bala Cynwyd	0.67	0.06
Penn Internal Medicine Woodbury Heights	0.46	0.002
Penn Family Medicine Unionville	0.42	0.11
Penn Presbyterian Internal Medicine	0.52	0.001
Interaction of Influenza Season & Practice Group		
Year 1 x Intervention practice group	Ref	
Year 2 x Intervention practice group	1.08	0.38

© 2018 Kim RH et al. *JAMA Network Open*.

Year 3 x Intervention practice group	1.52	0.0001
Intercept	0.30	<.0001

*GEE Model is clustered by primary care physician

**Main outcome measure highlighted in blue

© 2018 Kim RH et al. *JAMA Network Open*.

eTable 7. Regression Table for Difference-in-Differences Model Also Adjusted for Number of Patients Visits With the PCP in Each Season

Variable	Odds Ratio	P-Value
Influenza season		
Year 1	Ref	
Year 2	1.01	0.82
Year 3	0.98	0.72
Practice group		
Control	Ref	
Intervention	0.69	0.21
Patient age in years	1.02	<.0001
Patient sex		
Male	Ref	
Female	0.96	0.15
Patient race/ethnicity		
White non-hispanic	Ref	
Asian/East Indian	1.10	0.03
Black non-hispanic	0.82	<.0001
Hispanic	0.99	0.91
Other	0.83	<.0001
Patient insurance		
Private	Ref	
Medicare	1.50	<.0001
Medicaid	1.41	<.0001
Patient charlson comorbidity index	1.03	<.0001
Patient median household income	1.00	<.0001
Intervention practice site		
Penn Internal Medicine Radnor	Ref	
Penn Internal Medicine University City	1.11	0.65
Penn Center for Primary Care	1.25	0.32
Control practice site		
Penn Internal Medicine Cirigliano Practice	Ref	
Penn Internal Medicine Media	0.55	0.006
Penn Internal Medicine J. Edwin Wood Clinic	0.85	0.48
Penn Family Medicine Kennett	0.57	0.06
Penn Medicine Bala Cynwyd	0.61	0.02
Penn Internal Medicine Woodbury Heights	0.39	<.001
Penn Family Medicine Unionville	0.37	0.08
Penn Presbyterian Internal Medicine	0.49	<.001
Interaction of Influenza Season & Practice Group		
Year 1 x Intervention practice group	Ref	

© 2018 Kim RH et al. *JAMA Network Open*.

Year 2 x Intervention practice group	1.05	0.54
Year 3 x Intervention practice group	1.50	<.001
Number of PCP Visits	1.55	<.0001
Intercept		

*GEE Model is clustered by primary care physician

**Main outcome measure highlighted in blue

© 2018 Kim RH et al. *JAMA Network Open*.

eTable 8. Regression Table for Difference-in-Differences Model Using Generalized Linear Mixed Effects With Physician Random Effects Nested Within Practice Random Effects

Variable	Odds Ratio	P-Value
Influenza season		
Year 1	Ref	
Year 2	1.08	<.001
Year 3	1.06	0.01
Practice group		
Control	Ref	
Intervention	1.20	0.95
Patient age in years	1.02	<.0001
Patient sex		
Male	Ref	
Female	0.93	<.0001
Patient race/ethnicity		
White non-hispanic	Ref	
Asian/East Indian	1.09	0.05
Black non-hispanic	0.84	<.0001
Hispanic	1.08	0.20
Other	0.84	<.0001
Patient insurance		
Private	Ref	
Medicare	1.59	<.0001
Medicaid	1.47	<.0001
Patient charlson comorbidity index	1.05	<.0001
Patient median household income	1.00	1.00
Interaction of Influenza Season & Practice Group		
Year 1 x Intervention practice group	Ref	
Year 2 x Intervention practice group	1.01	0.76
Year 3 x Intervention practice group	1.53	<.0001
Intercept	0.20	0.30

*Glimix Model with physician random effects nested in practice random effects

**Main outcome measure highlighted in blue

© 2018 Kim RH et al. *JAMA Network Open*.

eFigure 1. Exclusions for the Patient Sample for the Seasonal Evaluation

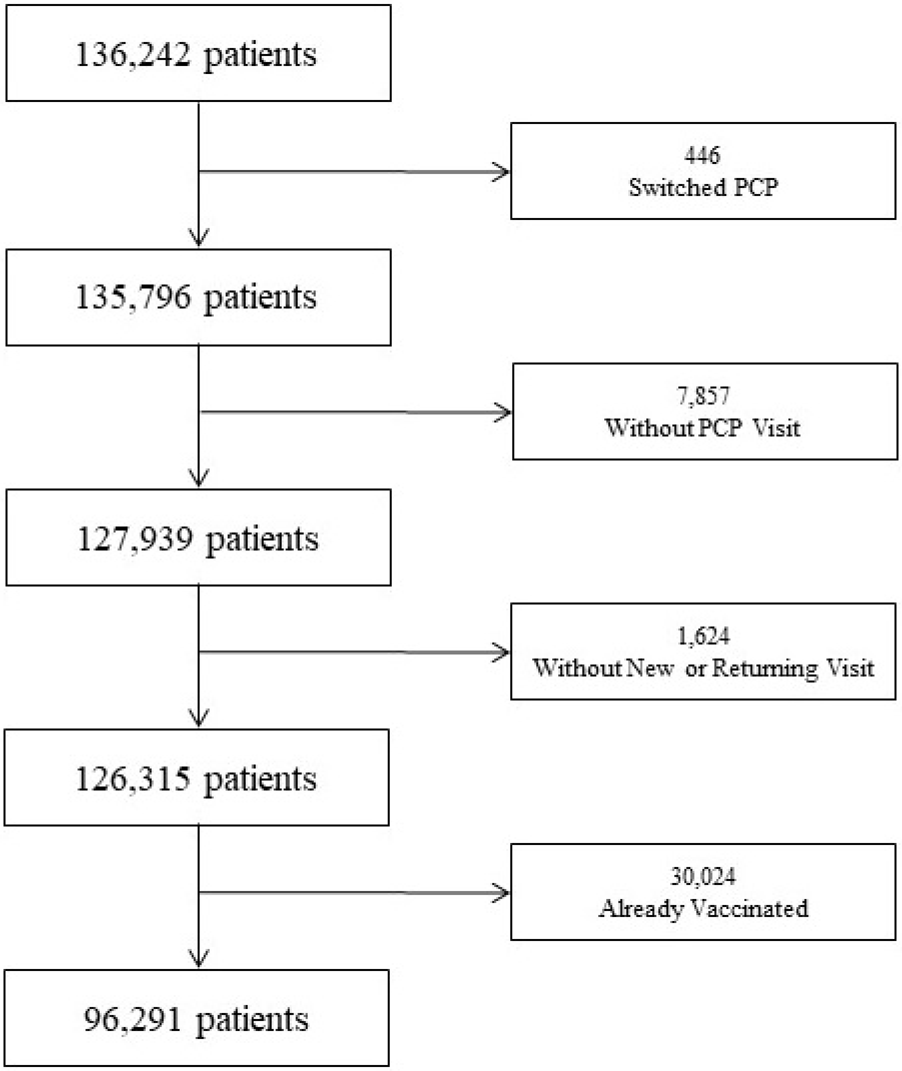

© 2018 Kim RH et al. *JAMA Network Open*.

eFigure 2. Exclusions for the Patient Visit Sample to Define the First Visit With the Primary Care Physician

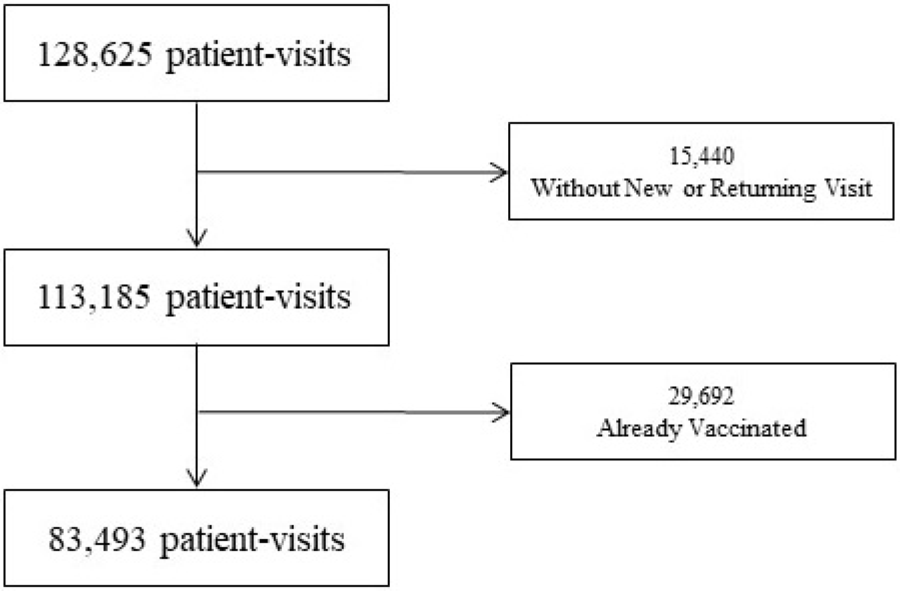

© 2018 Kim RH et al. *JAMA Network Open*.

eFigure 3. Active Choice Intervention Delivered Through a Best Practice Alert in EPIC

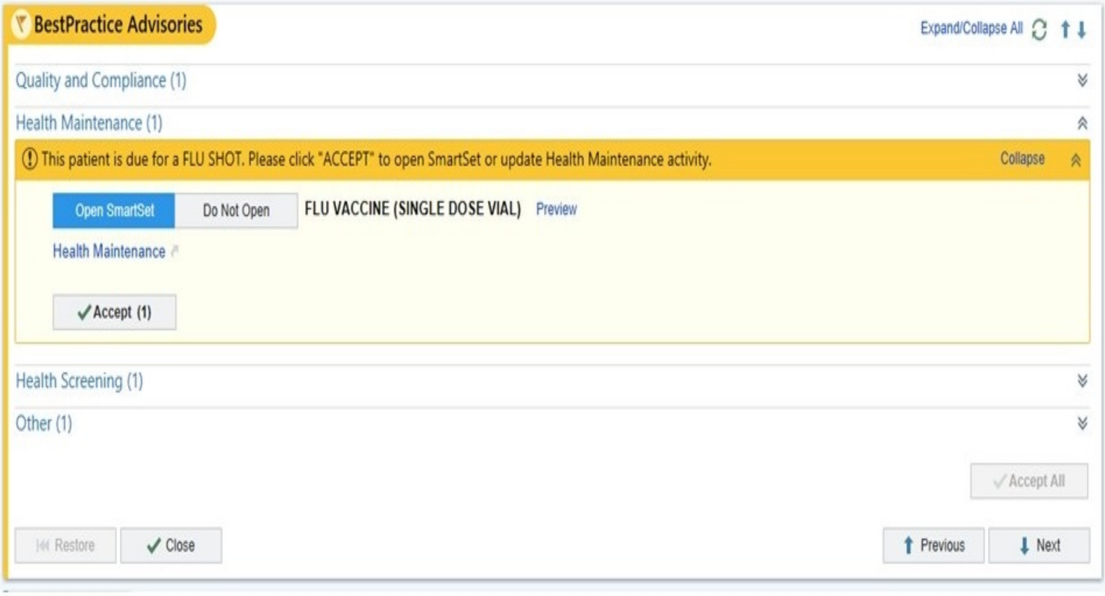

© 2018 Kim RH et al. *JAMA Network Open*.
